# Survey on the levels of 25-hydroxy vitamin D and bone metabolic markers and evaluation of their correlations with osteoporosis in perimenopausal woman in Xi’an region

**DOI:** 10.1371/journal.pone.0180366

**Published:** 2017-07-07

**Authors:** Ping Zhou, Jian Hu, Ping Xi, Ning Zhang, Bo Yang, Jie Zheng, Xiaoqin Wang

**Affiliations:** 1 The Laboratory Department of Xi'an Jiaotong University Medical College First Affiliated Hospital, Xi'an, Shaanxi, China; 2 The Endocrinology Department of Xi'an Jiaotong University Medical College First Affiliated Hospital, Xi'an, Shaanxi, China; 3 The Clinical Research Center of Xi'an Jiaotong University Medical College First Affiliated Hospital, Xi'an, Shaanxi, China; University of Alabama at Birmingham, UNITED STATES

## Abstract

It has been accepted that vitamin D (VD) plays an important role in bone metabolism. However, the levels of VD in people of different regions are quite different and there is still no final conclusion on the significant correlation between VD and osteoporosis. 245 cases of peri-menopausal women were collected to study the relationship between VD and osteoporosis in western China. The mean value of 25-hydroxyvitamin D for the participants was 14.39 ng/mL. The average values of parathyroid hormone (PTH), calcium (Ca) and phosphorus (P) were 47.62 pg/mL, 2.26 mmol/L and 1.18 mmol/L, respectively. The average value of bone mineral density (BMD) in lumbar vertebrae was -1.20 SD and that in femoral neck was -0.04 SD. Compared with normal group, PTH of VD deficiency group was significantly increased (P < 0.05), Ca was remarkably decreased (P < 0.01) while the BMD between these two groups showed no significant difference (P > 0.05). VD was in positive correlation with the age (P < 0.01) and Ca (< 0.01) of the participants, negative correlation with PTH (P < 0.01) while no significant correlation with the BMD of lumbar vertebrae and femoral neck (P > 0.05). The risk factors resulting in the occurrence of osteoporosis in the lumbar vertebrae of the participants covered Ca increase (OR = 66.247, P<0.05), age growth (OR = 1.194, P<0.01) and menopause (OR = 2.285, P<0.05). This study has found that the status of VD deficiency showed no significant correlation with the level of BMD, which hinted that independent measurement of the bone metabolic markers, including Ca, P, VD and PTH, was difficult to accurately reflect the status of BMD in peri-menopausal women of this region. It’s necessary to combine multi-site bone scanning to diagnose the patients’ status of osteoporosis so as to provide reasonable guidance for early clinical prevention and treatment.

## Introduction

In recent years, researchers have conducted intensive investigations and discussions on the important role of vitamin D in bone metabolism. VD deficiency may lead to rickets in children and chondromalacia in adults, and VD deficiency in elder people and menopausal women will result in bone loss and even the occurrence of osteoporosis and bone fractures [1,2]. Along with the increasing degree of aged phenomenon, the number of people may suffer the prevalence risk of osteoporosis is growing year by year. Timely discovery of VD deficiency or insufficiency and appropriate VD supplement are important means for the prevention and treatment of osteoporosis [3,4]. However, the levels of VD in people from different regions of the world differ greatly due to the influence of these factors, including race, gender, age, lifestyle, eating habits and sunlight exposure of residential region [5]. Recent studies have showed that the levels of VD in people of some regions show no significant correlation with osteoporosis [6–8]. Moreover, meta-analysis has indicated that independent VD supplement presents no therapeutic effect on people with osteoporosis [9], which hints that the effect of VD in bone metabolism and the incidence of osteoporosis in different regions and ethnic groups may exist differences. In addition, influenced by menopausal factors, perimenopausal women’ endocrine environments are undergoing significant changes and they are easy to suffer from osteoporosis [4]. Therefore, this study is expected to analyze the relation between the indexes, including VD, in perimenopausal women of this region and osteoporosis, and define the risk factors resulting in the occurrence of osteoporosis to perimenopausal women of this region so as to provide clinical guidance for effective prevention and treatment of osteoporosis by investigating the incidence of osteoporosis in perimenopausal women at the age of 40–60 in Xi'an region of Shaanxi province, and the distribution of calcium-phosphorus metabolic indexes including VD, PTH, Ca and P.

## Materials and methods

### Subjects

This study is a retrospective cohort study. The study protocol (S1 File) was in compliance with the Declaration of Helsinki, following written informed consent and approved by the ethics committee of Xian Jiaotong University First Affiliated Hospital (KYLLSL-2015-002). Perimenopausal women at the age of 40–60 consulting at the Outpatient Department of the First Affiliated Hospital of Xi'an Jiaotong University from April 2013 to October 2015 were collected. Underlying diseases, including autoimmune disease, cardiovascular and cerebrovascular disease, tumorous disease, endocrine disease, chronic kidney disease and chronic bone disease (except for osteoporosis or hyperostosis) were excluded by inquiring their past medical histories meanwhile combining with the results of their recent routine blood and urine tests, liver and kidney function biochemistry, tumor markers, thyroid function, rheumatism and immunology examination and other tests. In addition, patients with osteoporosis once taken any anti-osteoporosis medicines or nutrition supplement preparations, such as drug-induced VD and Ca, within six months were excluded.

### Data collection

General information, including the participants' age, height, weight, BMI and menstrual status, was collected and recorded in Patient’s Information Form for each subject. LEXXOS dual-energy x-ray absorptiometry (DMS, France) was used to determine the bone mineral density (BMD, g/cm^2^) at the parts of lumbar L1~L4 and femoral necks of the participants. T values (BMD, SD) of the participants’ BMD were calculated based on the reference value of BMD of young healthy women in Asian region to decide whether the participants were suffering from osteoporosis, of which T ≥-1 represented normal BMD, -2.5<T<-1 represented BMD decrease and T ≤-2.5 represented osteoporosis. Fasting venous blood collection was conducted to all participants at 7~8 in the morning for serum separation. Within 6 hours after blood collection, Cobas 8000 electrochemical luminescence immunity analyzer and auxiliary reagent (Roche, Switzerland) were used to determine the levels of the participants’ 25(OH)D and PTH. Labospect 008 full automatic biochemical analyzer (Hitachi, Japan) and auxiliary reagent were used to determine the levels of the participants’ Ca and P. All the test results for each subject were also recorded in the Patient’s Information Form for subsequently data analysis.

### Statistical analysis

In this study, SPSS 19.0 software was used to establish database and conduct all statistical analysis. All measurement data conforming to normal distribution was expressed as mean ± standard deviation. ANOVA variance analysis was adopted for the differences between groups. Pearson correlation analysis was adopted for the correlation between data to calculate the correlation coefficients between data. Logistic binary regression analysis was used for the prediction of independent risk factors for diseases.

## Results

### Participants’ basic information and test indexes

Subjects participating in this survey were mostly the patients consulting at the Outpatient of Rheumatism and Orthopedics Department of the First Affiliated Hospital of Xi'an Jiaotong University. By means of tracking these patients’ diagnosis consultation, inquiring their past medical histories and consulting their recent auxiliary examination results, 352 cases were included in the participants after excluding the patients with autoimmune disease, cardiovascular and cerebrovascular disease, tumorous disease, endocrine disease, chronic kidney disease and chronic bone disease (except for osteoporosis or hyperostosis) from the above ones. 42 cases of the participants once taken anti-osteoporosis medicines or supplementary preparations, such as VD and Ca, in recent period were excluded. Besides, 65 cases of the participants were also excluded due to the deficiency of key data (age, menstrual status, 25(OH)D and BMD). Eventually, a total of 245 cases of participants were included in data analysis.

The average age of these 245 cases of participants was within 49.5±5.5, of which a total of 167 cases were menopausal women, accounting for 68.2%. The average BMI of the participants was within 23.4±2.8 kg/m^2^, of which overweight ones (BMI>25.0) accounting for 26.9% (see Table 1). The BMI distributions of menopausal participants and premenopausal ones showed no significant difference (P > 0.05) (see Table 2).

**Table 1:** Participants’ basic information.

	Means±SD	Quartile(Q1-Q3)
Age	49.5±5.5	45.0–54.0
Weight	59.4±7.8	54.0–65.0
Height	159.2±4.8	156.0–162.0
BMI (kg/m^2^)	23.4±2.8	21.6–25.1
25(OH)D (ng/mL)	14.39±6.63	9.98–18.15
PTH (pg/mL)	47.62±16.94	37.46–53.04
Ca (mmol/L)	2.26±0.10	2.19–2.31
P (mmol/L)	1.18±0.44	1.05–1.24
BMD (Lumbar, g/cm^2^)	0.83±0.16	0.73–0.96
BMD (Lumbar, SD)	-1.20±1.43	-2.25–0.10
BMD (Femoral neck, g/cm^2^)	0.88±0.14	0.79–0.98
BMD (Femoral neck, SD)	-0.04±1.14	-0.80–0.80

**Table 2:** Correlation between postmenopausal and premenopausal participants' indexes (including 25(OH)D) and BMD (ANOVA).

	Premenopausal [Mean(SD)]	Postmenopausal [Mean(SD)]	*P*-value
Number	78	167	/
Age	47.2(4.7)	50.6(5.4)**	0.000
Weight	59.2(7.8)	59.5(7.8)	0.776
Height	159.7(5.3)	159.0(4.6)	0.333
BMI	23.2(2.8)	23.5(2.8)	0.433
25(OH)D (ng/ml)	13.40(6.84)	14.88(6.55)	0.093
PTH (pg/mL)	50.19(20.82)	46.44(14.75)	0.131
Ca (mmol/L)	2.25(0.11)	2.26(0.09)	0.217
P (mmol/L)	1.13(0.15)	1.20(0.53)	0.256
BMD (L1–L4,g/cm^2^)	0.87(0.15)	0.82(0.16)*	0.033
BMD (L1–L4,SD)	-0.86(1.44)	-1.36(1.40)*	0.011
BMD (Femoral neck,g/cm^2^)	0.90(0.16)	0.87(0.13)	0.099
BMD (Femoral neck,SD)	0.30(1.02)	-0.20(1.16)**	0.001

Note

* represents P<0.05

** represents P<0.01.

The average value of these 245 cases of participants’ 25(OH)D was within 14.39±6.63 ng/mL, of which 82.0% of participants’ 25(OH)D was lower than the lower limit of China's current reference range (20 ng/mL) [10], and 40.8% of participants’ 25(OH)D was below 12 ng/mL, conforming to China’s Judgment Standard for VD Deficiency [10]. 25(OH)D distribution of menopausal participants and premenopausal ones showed no significant difference (P > 0.05) (see Table 2).

The average value of 216 cases of participants’ PTH (29 cases were absent of this information) was within 47.62±16.94 pg/mL, of which 87.5% of participants’ PTH was within the reference range (16–65 pg/mL) Roche recommended (see Table 1). The average value of 234 cases of participants’ Ca (11 cases were absent of this information) was within 2.26±0.10 mmol/L, of which 94.0% of participants’ Ca level was within the range of China’s current reference interval (2.11–2.52 mmol/L) (see Table 1) and 96.8% of participants’ P level was within the range of China’s current reference interval (0.85–1.51 mmol/L) (see Table 1). The distribution of PTH, Ca and P in menopausal participants and premenopausal ones showed no significant difference (P > 0.05) (see Table 2).

The average value of BMD in the lumbar vertebrae of 245 cases of participants was within 0.83±0.16 g/cm^2^ and the average of T value was within -1.20±1.43 SD, of which participants with osteoporosis accounting for 21.2% and participants with osteopenia accounting for 33.5%. The average value of BMD in femoral neck was within 0.88±0.14 g/cm^2^ and the average of T value was within -0.04±1.14 SD, of which participants with osteoporosis accounting for 1.6%, and participants with osteopenia accounting for 19.6% (see Table 1). Of these participants, patients with osteoporosis in lumbar vertebrae were significantly more than the ones with osteoporosis in femoral neck, and T value of BMD in the lumbar vertebrae of 88.2% of participants was lower than it in femoral neck. T value of BMD in the lumbar vertebrae and femoral neck of menopausal participants was significantly lower than pre-menopausal ones (respectively, P < 0.05 and P < 0.01) (see Table 2).

### Comparison between the BMD of participants with different levels of 25(OH)D and other bone metabolic markers

According to the Recommended Standard under China Guide for Healthy Application of Vitamin D in Adult Skeleton, 245 cases of participants in this study were grouped on the basis that whether the judgment standards, 12 ng/mL and 20 ng/mL respectively, were in VD deficiency, and the differences between the participants’ ages, BMD and bone metabolism markers under different grouping basis were compared. Statistical results have indicated that whether in accordance with the classification standard of 12 ng/mL or 20 ng/mL, compared with high 25(OH)D level group, the ages of the participants of low 25(OH)D group were relatively low (P <0.01 and 0.05), PTH was relatively high (P <0.01 and 0.05) and Ca was relatively low (P average <0.01) while other indexes, including BMI, P and BMD, showed no significant difference (P > 0.05) (see Table 3). In addition, this study also compared the differences of above indexes in the participants of 25(OH)D<12 ng/mL group and 25(OH)D>20 ng/mL group. Statistical results have showed that significantly different indexes existing in the participants of these two groups still were age (P < 0.01), PTH (P < 0.01) and Ca (P < 0.01) while other indexes showed no significant difference (P > 0.05) (see Table 3).

**Table 3:** Correlation between various indexes of participants with different levels of 25(OH)D and BMD (ANOVA).

	25(0H)D						*P*-value VD<12 VS VD≥20
	Cut off: 12 ng/mL			Cut off: 20ng/mL			
	<12 ng/mL Mean(SD)	≥12 ng/mL Mean(SD)	*P*-value	<20 ng/mL Mean(SD)	≥20 ng/mL Mean(SD)	*P*-value	
Number	103	142	/	205	40	/	/
Age	48.4(5.8)	50.4(5.1) **	0.004	49.1(5.5)	51.5(4.8)*	0.011	0.002**
Weight	58.4(8.0)	60.2(7.5)	0.084	59.5(8.0)	58.9(6.6)	0.610	0.762
Height	159.5(4.7)	159.0(5.0)	0.479	159.3(4.8)	158.9(5.1)	0.665	0.530
BMI	23.0(3.1)	23.8(2.6)	0.033	23.5(2.9)	23.3(2.4)	0.771	0.545
PTH (pg/mL)	52.58(20.32)	44.21(13.20)**	0.000	48.93(17.76)	41.45(10.52)*	0.013	0.002**
Ca (mmol/L)	2.23(0.10)	2.28(0.10)**	0.000	2.25(0.09)	2.30(0.11)**	0.006	0.001**
P (mmol/L)	1.19(0.67)	1.17(0.14)	0.738	1.17(0.48)	1.20(0.15)	0.773	0.954
BMD (L1–L4,g/cm^2^)	0.843(0.150)	0.828(0.167)	0.473	0.838(0.154)	0.820(0.188)	0.537	0.447
BMD (L1–L4,SD)	-1.119(1.367)	-1.266(1.480)	0.432	-1.196(1.393)	-1.248(1.641)	0.835	0.636
BMD (Femoral neck,g/cm^2^)	0.860(0.156)	0.888(0.132)	0.134	0.878(0.144)	0.865(0.136)	0.605	0.850
BMD (Femoral neck,SD)	-0.080(1.096)	0.006(1.179)	0.621	-0.006(1.129)	-0.195(1.217)	0.340	0.585

Note

* represents P<0.05

** represents P<0.01.

### Correlation analysis of 25(OH)D with age, PTH, Ca, P and BMD

**Fig 1:**
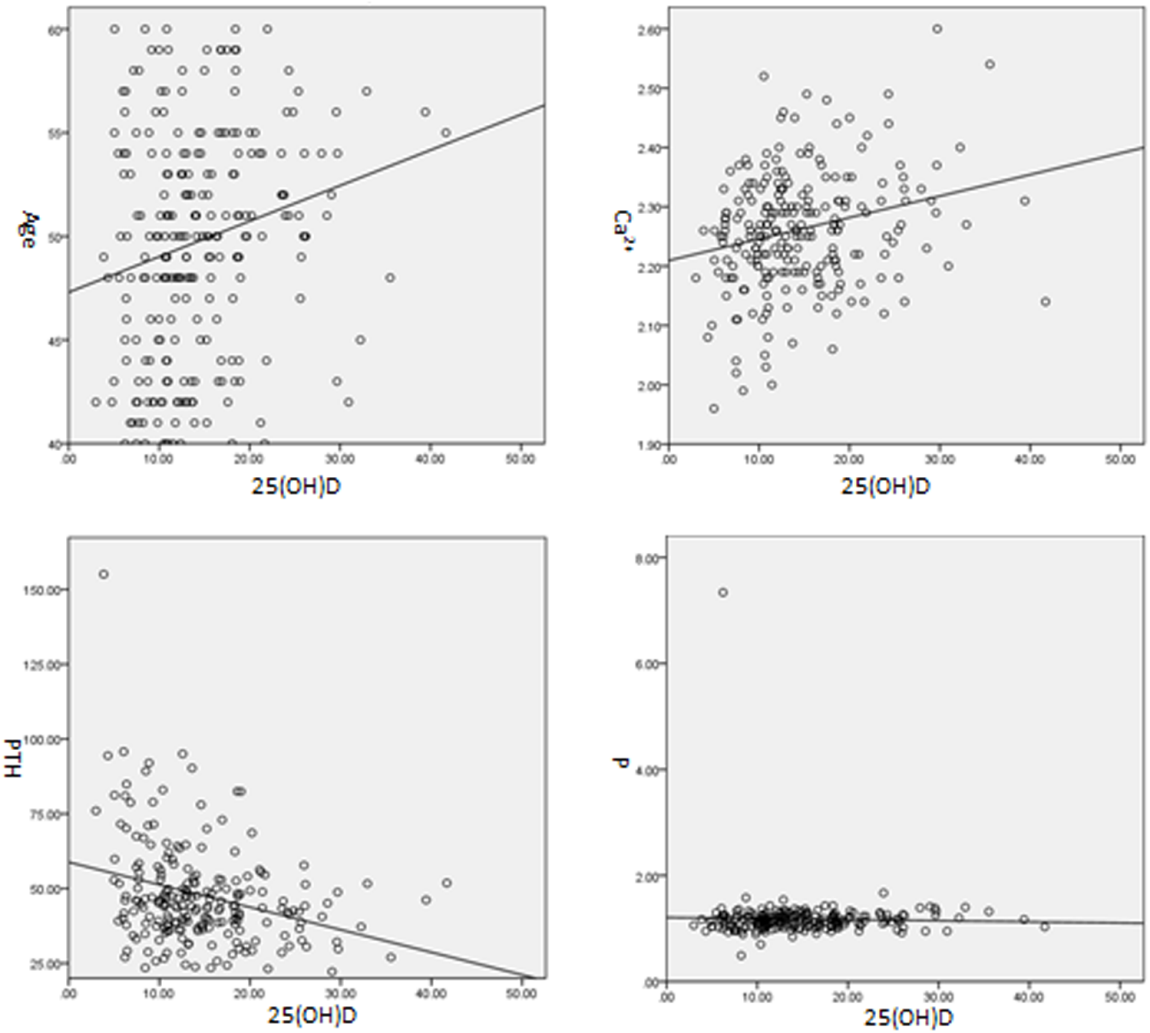
Correlation of 25(OH)D with Age, Ca, P and PTH.

**Fig 2:**
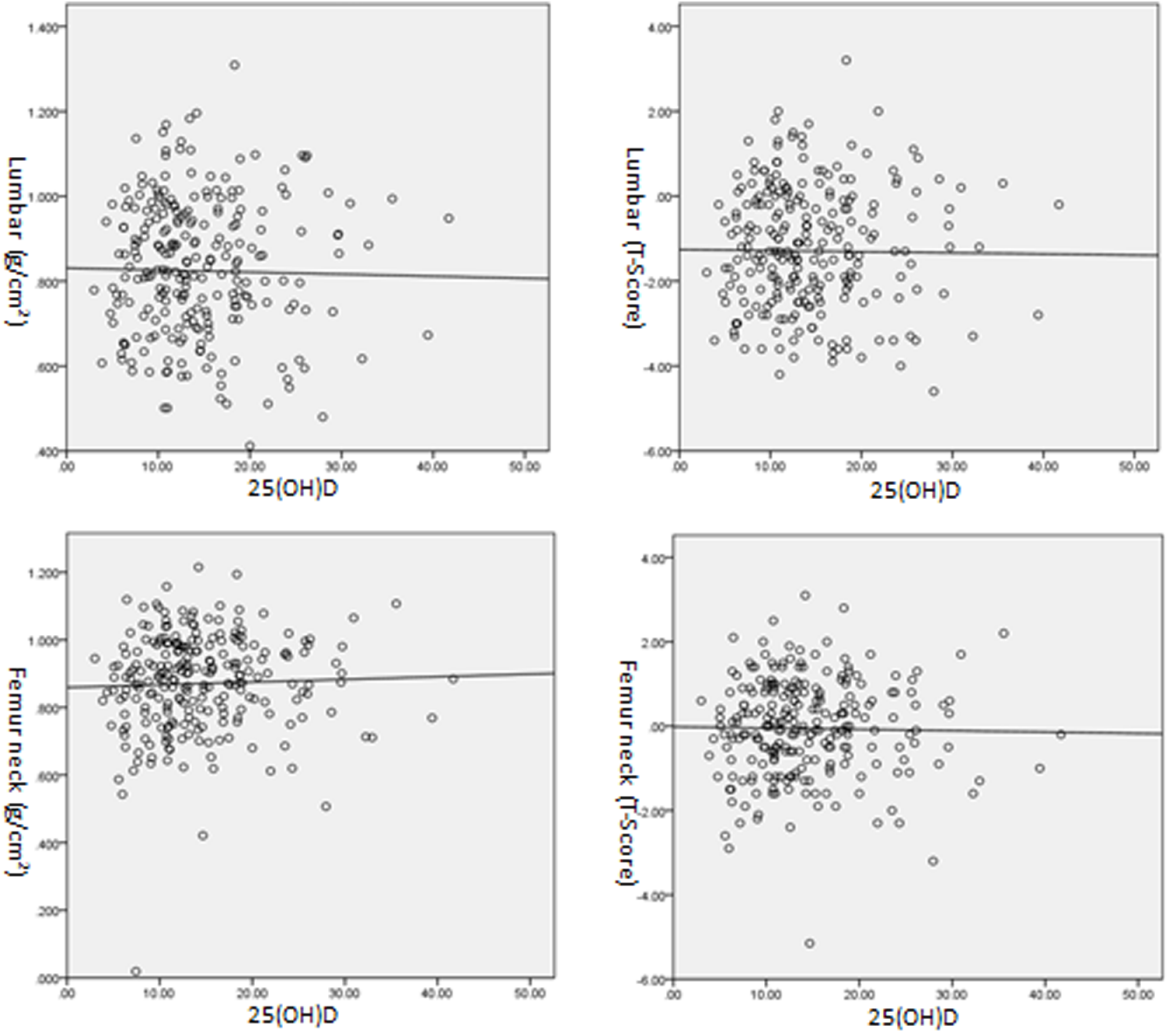
Correlation of 25(OH)D with BMD in lumbar vertebrae and femoral neck.

Study results from the comparison of bone metabolic markers of participants with different levels of 25(OH)D have showed that participants with different levels of 25(OH)D are only different in ages and the levels of PTH and Ca while show no significant difference in the levels of P and BMD. Therefore, this study further analyzed the correlation between 25(OH)D and these indexes. Studies have shown that 25(OH)D is in positive correlation with age (R = 0.202, P < 0.01) and Ca (R = 0.237, P < 0.001), negative correlation with PTH (R = 0.291, P < 0.001) while no significant correlation with P (R = 0.027, P = 0.690) (see Fig 1). In addition, 25(OH)D showed no significant correlation with the absolute value (R = -0.003, P = 0.964) and T value (R = 0.006, P = 0.930) of BMD in lumbar vertebrae as well as the absolute value (R = 0.068, P = 0.292) and T value (R = 0.025, P = 0.692) of BMD in femoral neck (see Fig 2).

### Risk evaluation of BMD decrease and the occurrence of osteoporosis

Risk factors from the application of Logistic binary regression analysis for BMD decrease and the occurrence of osteoporosis to participants indicated that the risk factors causing osteopenia in the lumbar vertebrae of participants covered blood Ca increase (OR = 18.178, P < 0.05) and age growth (OR = 1.168, P < 0.001). However, the risk factors causing the occurrence of osteoporosis in lumbar vertebrae covered menopause (OR = 2.285, P <0.05) in addition to blood Ca increase (OR = 66.247, P <0.05) and age growth (OR = 1.194, P <0.001). 25(OH)D, PTH, P and height had no significant relation with the decrease of BMD in participants’ lumbar vertebrae (see Table 4).

**Table 4:** Risk evaluation of BMD decrease in lumbar vertebrae and the occurrence of osteoporosis.

	Osteopenia		Osteoporosis	
	OR(95% CI)	*P*-Value	OR(95% CI)	*P*-Value
Ca	18.178(1.233–267.980)*	0.035	66.247 (2.530–1734.621)*	0.012
Age	1.168(1.106–1.233)**	0.000	1.194(1.113–1.280)**	0.000
Postmenopausal	1.657(0.964–2.847)	0.067	2.285 (1.079–4.838)*	0.031
Weight	0.946(0.913–0.979)**	0.002	0.936 (0.894–0.979)**	0.004
BMI	0.889(0.810–0.976)*	0.014	0.865 (0.767–0.975)*	0.018
Height	0.948(0.899–1.000)	0.052	0.959(0.899–1.022)	0.197
25(0H)D	0.994(0.957–1.032)	0.736	0.999(0.954–1.046)	0.963
PTH	0.998(0.982–1.014)	0.817	1.004(0.985–1.022)	0.695
P	0.989(0.542–1.804)	0.971	1.105(0.580–2.105)	0.762

Note

* represents P<0.05

** represents P<0.01.

Similar to lumbar vertebrae, the risk factors causing osteopenia in the femoral necks of participants covered blood Ca increase (OR = 84.345, P <0.05) and age growth (OR = 1.129, P <0.001). Menopause, 25(OH)D, PTH, and P had no significant relation with the decrease of BMD in femoral neck (P > 0.05) (see Table 5). In addition, the risk factors causing osteoporosis in femoral neck were not included because there were only 4 cases of participants suffering from osteoporosis in their femoral necks.

**Table 5:** Risk evaluation of osteopenia in femoral neck.

	OR(95% CI)	*P*-Value
Ca	84.345(2.906–2448.104)**	0.010
Age	1.129(1.059–1.205)**	0.000
BMI	0.777(0.679–0.889)**	0.000
Weight	0.885(0.838–0.935)**	0.000
Height	0.928(0.868–0.992)*	0.029
Postmenopausal	2.003(0.941–4.266)	0.072
25(0H)D	0.984(0.936–1.034)	0.523
PTH	1.000(0.981–1.020)	0.983
P	1.163(0.621–2.178)	0.637

Note

* represents P<0.05

** represents P<0.01.

## Discussion

Vitamin D is a fat-soluble vitamin, belongs to the sterol compounds. Among all the VD derivatives, activity of vitamin D2 and D3 is the highest. In organisms, vitamin D has no biological activity before the formation of 25(OH)D and 1,25(OH)_2_D after hydroxylation in certain cells of the liver, kidney and other organs. Moreover, previous study reported that, in addition to animal liver and kidney, novel VD pathways were also found in skin and adrenal gland. VD derivatives generated through these pathways might act as hormones in the regulation of variety of endocrine activities in human body [11, 12, 13]. 25(OH)D, a precursor of 1,25(OH)_2_D, is the main storage form of vitamin D in the human body and is capable for detecting the VD in the laboratory. Namely, 25(OH)D can be used to determine total VD in human body.

The source of VD in human body mainly is from two ways, of which one is the direct generation of skin by ultraviolet radiation and the other is exogenous intake from food. In view that modern people have different sunlight exposure and eating habits, the content of VD in people enjoying different latitudes, regions, dressing habits and eating habits may be significantly different [14,15]. Current studies generally consider that VD plays an important role in regulating calcium balance and bone metabolism. Studies have shown that Ca absorption decreases when VD is in deficiency, which accordingly causes the increase of secondary parathyroid hormone, accelerates the maturity of osteoclasts and further leads to bone loss. Particularly for the elder people, the decrease of VD level leads to the declining function of skeletal muscle, and thereby increasing the risk of falls and fractures [1,2]. The study of Holic 2005 has put forward that it’s called VD deficiency when VD in body blood is below 20 ng/ml, relative VD deficiency when VD in body blood is within 21 ~ 29 ng/ml and VD sufficiency when VD in body blood is above 30 ng/ml [16]. Currently, the judgment standards used in China take VD above 30 ng/ml as the best level, below 20 ng/ml as VD insufficiency while VD below 12 ng/ml is diagnosed as VD deficiency [10]. According to the above judgment standards in China, VD level in Chinese people is generally low and the rates of adult VD deficiency in different regions are all above 50% [17–19]. Therefore, China’s Guide for Healthy Application of Vitamin D in Adult Skeleton in 2014 pointed out that for people who are in VD insufficiency, deficiency and not easy to supplement VD from food or sunlight and other physiologic ways, they should make appropriate daily VD supplement to prevent the occurrence of calcium deficiency, osteoporosis and other diseases [10].

In this study, the 25(OH)D levels of participants collected from the perimenopausal women at the age of 40–60 consulting at the Outpatient Department of the First Affiliated Hospital of Xi'an Jiaotong University presented the status of significant deficiency, of which 40.8% of participants’ 25(OH)D levels were relatively insufficient (below 20 ng/mL), 40.8% of participants were seriously deficient (below 12 ng/mL) and its average value was lower than the VD level (16.38 ng/ml) of people in China’s Lanzhou region which enjoyed similar latitude, race, dressing habits and eating habits [6]. So far, related reports on VD levels of perimenopausal women in Xi'an region have not been covered. However, the result of a survey on VD in pregnant women in Xi'an in 2012 has showed that the average value of VD in pregnant women is 15.4 ng/mL [20], which is close to the VD level of female participants at the age of 40–60 in this study.

Previous researchers considered that as an important regulatory factor assisting the absorption of Ca and P, VD level in serum was in positive correlation with the levels of Ca and P while negative correlation with PTH. When VD is in significant deficiency, the absorption of Ca and P in human body is insufficient and the levels of blood Ca and P may be lower than the normal level, which will cause secondary parathyroid hyper-function. The increase of PTH affects bone deposition and further leads to the decrease of BMD, which increases the incidence risk of osteoporosis. Lots of studies have shown that VD deficiency has an important relation with Ca and P metabolic disorder, bone loss and BMD decrease [21–27]. However, from the analysis of other indexes that may be associated with VD in this study, it’s found that although most of participants (82.0%) were in VD insufficiency or deficiency, the distribution of their PTH and Ca was within normal reference range (normal PTH and Ca are 87.5% and 94.0%, respectively), and the proportion of participants with osteoporosis was not high (participants with osteoporosis in lumbar vertebrae accounting for 21.2% and participants with osteoporosis in femoral neck only accounting for 1.6%). Further analysis has found that compared with participants in normal VD, the participants suffering VD insufficiency or deficiency showed differences in ages, PTH and Ca while their P, BMD and other indexes showed no significant difference. Participants’ VD level was in positive correlation with Ca, negative correlation with PTH and no correlation with P and BMD of lumbar vertebrae and femoral neck. The findings of this study is similar to the study on Lanzhou region, participants’ VD level is related to P while in no significant correlation with Ca, and their VD showed no significant correlation with the occurrence of BMD and osteoporosis [6]. In addition, studies from Beijing have also found that the distributions of VD in postmenopausal women with normal bone mass and the ones with osteoporosis showed no significant difference and the participants’ BMD presented no significant correlation with VD [8]. In a large number of related foreign studies, South Korean studies have put forward that though VD showed certain correlation with BMD of male participants while relatively low correlation with BMD of female participants [28]. The surveys of Kuchuk et al on VD, BMD and PTH in postmenopausal women from 29 countries around the world have indicated that these three indexes had no significant correlation [29]. Meta-analysis, published in the journal Lancet in 2014, has proposed that VD supplement can’t improve the BMD of ordinary healthy adults [9]. Above studies have shown that the relations between VD and Ca, P metabolism and BMD, in people from different regions and races and enjoying different habits, are very complicated, and numerous genetic factors and non- genetic factors may affect the relation between VD and bone metabolism. Therefore, this study is of high clinical value for defining the correlation between the levels of bone metabolic markers (such as VD and PTH) and BMD of perimenopausal women in Xi'an region, and guiding early clinical diagnosis and treatment of osteoporosis.

In this study, from further analysis of the risk factors causing the occurrence of osteopenia and osteoporosis, it’s been found that blood Ca increase (but within the normal range) and age growth are the common risk factors for osteopenia and osteoporosis in lumbar vertebrae; Menopause is only the risk for the incidence of osteoporosis but has no relation with osteopenia; In addition, the levels of VD, PTH and P have no significant correlation with the BMD of participants’ lumbar vertebrae. The risk factors causing osteopenia in the femoral necks of participants also cover blood Ca increase and age growth while menopause, VD, PTH and P show no significant correlation with the BMD of participants’ femoral necks. This result has further proved that these two bone metabolic markers, VD deficiency and PTH increase, which were previously thought as important risk factors for osteoporosis, show no significant correlation in the pathogenic process of osteoporosis in perimenopausal women of this region. Simple VD deficiency or slight PTH increase can’t be used as the diagnostic basis for osteopenia in perimenopausal women of this region.

**Fig 3:**
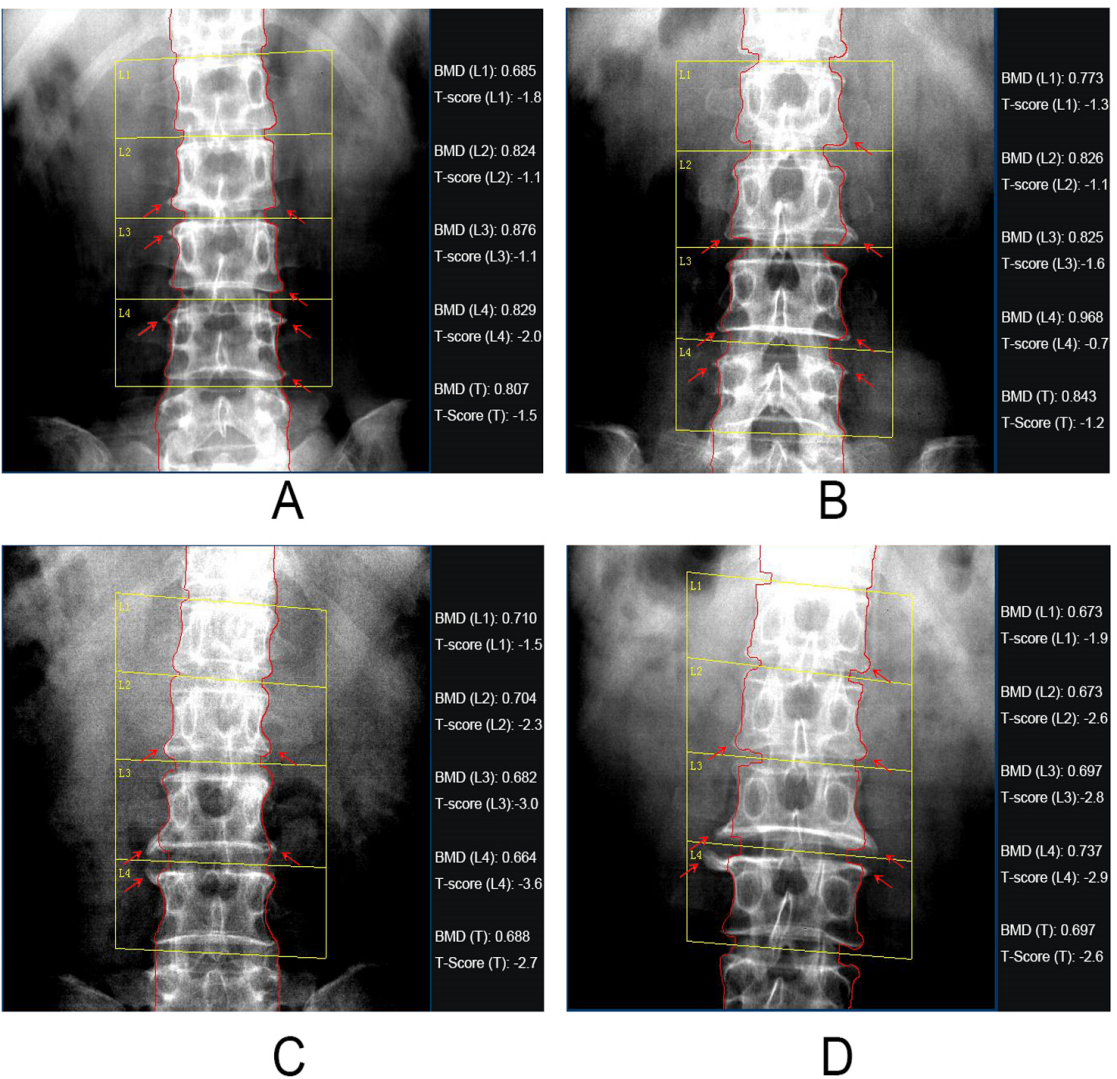
Participants’ X-ray films of lumbar vertebrae. 4 participants were selected. Two participants, A and B were patients with BMD decrease. The other two participants, C and D, were patients with osteoporosis. X-ray films were shot by LEXXOS dual-energy X-ray absorptiometry. The status of BMD in the lumbar vertebrae of participants, including the respective and total (T) actual measured value and corresponding T value (T—score) of BMD in their lumbar L1-L4, was marked on the right side of each X-ray film. The red contour lines around vertebral body indicate the outer contour of vertebral body when measuring BMD, and the red arrows indicate the status of bone spur formed by the participants’ hyperostosis.

For the possible mechanism of the above distribution status presented by the participants’ VD, bone metabolic markers and BMD in this study, we consider that intestinal absorption of Ca and P is decreased under the condition of VD deficiency while the decline of Ca and P levels in blood can stimulate slight increase of PTH secretion. Upregulated PTH level accelerates old bone absorption and bone salt dissolution, and releases Ca and P to supplement the deficiency of blood Ca and P. However, the effect of increased PTH on kidney P excretion is strengthened, which makes blood P decline to the normal range. Therefore, the participants show the status of VD deficiency, slight PTH increase, slight blood Ca increase and unobvious change of blood P. Under normal circumstances, bone salt can’t make effective deposition due to the constant effect of the above status on skeleton. It eventually leads to bone demineralization and presents changes in BMD decline. However, in China, hyperostosis is mostly common in elder people and its common symptoms cover abnormal bone accumulation in Lumbar vertebrae and femoral neck, and increasing BMD heterogeneity (see Fig 3). In this study, the participants’ skeletons presented bone loss and decreasing BMD reference value under the action of VD deficiency and PTH increase. However, due to the influence of hyperostosis, the participants’ skeletons showed the combination of osteoporosis and hyperostosis, which eventually alleviated the degree of BMD decrease in these participants and even showed normal BMD. In addition to the explanation above, patients involved in this study were all perimenopausal women. So it can't exclude the possibility that the patients had taken estrogen containing drugs to alleviate perimenopausal syndrome. This might cause the inconsistence between VD level and bone mineral density. Meanwhile, there are many drugs used in the treatment of perimenopausal syndrome in China, including quite a number of traditional Chinese medicines, which may contain plant or animal estrogen. The component of traditional Chinese medicines is extremely complex. So we can only know whether the patient had taken any traditional Chinese medicines through inquiry, however, it’s difficult to obtain specific information about the prescription and components of the drugs. Whereas, it has been confirmed that estrogen has the function of relieving osteoporosis in postmenopausal women. So, the impact of estrogen to the bone mineral density in this study needs to be investigated in our future work.

The theory that Vitamin D and calcium play an important role in bone metabolism in humans has been widely recognized by academia. In this manuscript, after the investigation of VD, PTH, calcium, phosphorus, and bone mineral density index in perimenopausal women, we found that there was no significant correlation between VD level and bone mineral density. This seems to question that VD played a role in bone metabolism. However, in fact this study is not challenging the prevailing academic identity theory, but hopes to put forward that this regional perimenopausal women have a variety of physiological and pathological changes, including lack of VD, osteoporosis and bone hyperplasia, etc. Therefore we can’t assess patient's bone metabolism level simply rely on Ca, P, PTH and VD, the level of bone metabolic markers and different parts of the lumbar spine and femoral neck bone mineral density index should also be analyzed to conclude more rational results. As to the explanation for the insignificant correlation between bone mineral and VD level of patients. We think there must be some unrevealed mechanisms with corresponding effect, which make the bone mineral density of patient not dropped significantly in the case of VD deficiency. For instance, co-existence of osteosis and osteoporosis was detected during bone X-ray scanning, it can partly explain the insignificant correlation between bone mineral density and VD level. However, as we mentioned above, there must be some other mechanisms which have not been described yet. So, our further study will focus on revealing these mechanisms.

To sum up, this study considers that VD deficiency in perimenopausal women of Xi'an region is very common and there is no significant correlation between VD deficiency and the level of BMD. Therefore, simple measurement of the levels of bone metabolic markers, such as Ca, P, VD and PTH, can’t really reflect the status of perimenopausal women's BMD in this region. It’s necessary to combine bone markers with bone scanning to diagnose the patients’ status of bone loss. Therefore, this study is of great significance to reasonably guide early clinical intervention of osteoporosis treatment and the occurrence of related fractures.

## Supporting information

S1 File The protocol for this study. (PDF)

S2 File The strobe checklist for this study. (DOCX)
