# Supplementary material for: Survey on the levels of 25-hydroxy vitamin D and bone metabolic markers and evaluation of their correlations with osteoporosis in perimenopausal woman in Xi’an region
Source: PLoS One. 2017 Jul 7;12(7):e0180366. doi: 10.1371/journal.pone.0180366 (PMC5501542; doi:10.1371/journal.pone.0180366)
Supplement: S1 File — (PDF) [file pone.0180366.s001.pdf]

Survey on the Levels of 25-hydroxy Vitamin D and Bone Metabolic Markers and Evaluation of Their Correlations with Osteoporosis in Perimenopausal Woman in Xi’an Region

Survey protocol:

1.Peri-menopausal women aged 40-60 years who visit the outpatient clinic for physical examination and consultation will be screened in cooperation with Department of Endocrinology and Department of Rheumatology of The First Affiliated Hospital of Xi'an Jiaotong University: patients with endocrine system diseases, autoimmune diseases, cardiovascular and cerebrovascular diseases, tumors and other underlying diseases or osteoporosis patients that have been treated with anti-osteoporosis drugs previously will be excluded after the inquiry of past medical history, general physical examination and routine auxiliary examinations, and the patients who have not been treated with medicinal vitamin D, Ca or other nutritional supplements in the past six months will be recruited, and they will sign the Informed Consent Form and complete the survey questionnaire.

2.The survey questionnaire involves the patients' height, weight, age, occupation, smoking, alcoholic drinking and other bad habits, menopause or not, suffering from osteoporosis or not, history of bone fracture in the past three years, use of hormone replacement therapy, use of oral contraceptives, corticosteroids, thyroid hormones and psychotropic drugs, and other questions.

3.For any subject that has completed the survey questionnaire, 3-5 mL venous blood will be collected from them under a fasting state during 07:00 a.m.-08:00 a.m. in the next morning into a blood collection tube containing no additive, and then the subject’s bone mass density (BMD, g/cm^2) of lumbar vertebrae L1-L4 and femur neck will be determined using the LEXXOS Dual Energy X-rays absorptiometry bone densitometer (DMS, France), and the subject’s T-score of bone mass density (BMD, SD) will be calculated based on the bone mass density of young women in Asia as the baseline value, so as to judge if the subject has osteoporosis or not.

4.For the venous blood collected above, allow to stand at room temperature for one hour, centrifugate at 4000 rpm for 10 min to separate serum, and divide into aliquots in three EP tubes. The serum 25(OH)D and PTH levels in one tube will be determined using Roche Cobas 8000 electrochemistry luminescence immunity analyzer and its supporting reagent; the serum Ca and P levels in another tube will be determined using Hitachi Labospect 008 Automatic Analyzer and its supporting reagent (Wako, Japan); and the rest one will be frozen in a -80 ℃ refrigerator for future use.

5. All the measurement data mentioned above will be entered into the SPSS 19.0

software and statistical analysis on the data will be performed. All the measurement results will be expressed as mean ± standard deviation (M ± SD), and the ANOVA (analysis of variance) will be performed for the subjects' inter-group differences; data correlation will be determined using Pearson correlation analysis, and the disease-related risk factors will be evaluated and summarized using Binary Logistic Regression. All the data will be entered into word forms for retention.
