# Supplementary material for: Survey on the levels of 25-hydroxy vitamin D and bone metabolic markers and evaluation of their correlations with osteoporosis in perimenopausal woman in Xi’an region
Source: PLoS One. 2017 Jul 7;12(7):e0180366. doi: 10.1371/journal.pone.0180366 (PMC5501542; doi:10.1371/journal.pone.0180366)
Supplement: S2 File — (DOCX) [file pone.0180366.s002.docx]

STROBE Statement—checklist of items that should be included in reports of observational studies

|  | Item No. | Recommendation | Page  No. | Relevant text from manuscript |
| --- | --- | --- | --- | --- |
| **Title and abstract** | 1 | (*a*) Indicate the study’s design with a commonly used term in the title or the abstract | 1 | Survey on the Levels of 25-hydroxy Vitamin D and Bone Metabolic Markers and Evaluation of Their Correlations with Osteoporosis in Perimenopausal Woman in Xi’an Region |
|  |  | (*b*) Provide in the abstract an informative and balanced summary of what was done and what was found | 2-3 | This study has found that the status of VD deficiency showed no significant correlation with the level of BMD, which hinted that independent measurement of the bone metabolic markers, including Ca, P, VD and PTH, was difficult to accurately reflect the status of BMD in peri-menopausal women of this region. |
| Introduction | | | |  |
| Background/rationale | 2 | Explain the scientific background and rationale for the investigation being reported | 3-4 |  |
| Objectives | 3 | State specific objectives, including any prespecified hypotheses | 4 |  |
| Methods | | | |  |
| Study design | 4 | Present key elements of study design early in the paper | 4 | This study is a retrospective cohort study. |
| Setting | 5 | Describe the setting, locations, and relevant dates, including periods of recruitment, exposure, follow-up, and data collection | 4-6 | Perimenopausal women at the age of 40-60 consulting at the Outpatient Department of the First Affiliated Hospital of Xi'an Jiaotong University from April 2013 to October 2015 were collected. All the test results for each subject were also recorded in the Patient’s Information Form for subsequently data analysis. |
| Participants | 6 | (*a*) *Cohort study*—Give the eligibility criteria, and the sources and methods of selection of participants. Describe methods of follow-up  *Case-control study*—Give the eligibility criteria, and the sources and methods of case ascertainment and control selection. Give the rationale for the choice of cases and controls  *Cross-sectional study*—Give the eligibility criteria, and the sources and methods of selection of participants | 4-5 | 245 cases of peri-menopausal women were recruited to this study to study the relationship between VD and osteoporosis. |
|  |  | (*b*) *Cohort study*—For matched studies, give matching criteria and number of exposed and unexposed  *Case-control study*—For matched studies, give matching criteria and the number of controls per case | N/A |  |
| Variables | 7 | Clearly define all outcomes, exposures, predictors, potential confounders, and effect modifiers. Give diagnostic criteria, if applicable | 5 | Levels of the participants’ 25(OH)D, PTH, Ca and P |
| Data sources/ measurement | 8* | For each variable of interest, give sources of data and details of methods of assessment (measurement). Describe comparability of assessment methods if there is more than one group | N/A |  |
| Bias | 9 | Describe any efforts to address potential sources of bias | N/A |  |
| Study size | 10 | Explain how the study size was arrived at | 6 | By means of tracking these patients’ diagnosis consultation, inquiring their past medical histories and consulting their recent auxiliary examination results, 352 cases were recruited. Eventually, a total of 245 cases of participants were included in data analysis. |

Continued on next page

| Quantitative variables | 11 | Explain how quantitative variables were handled in the analyses. If applicable, describe which groupings were chosen and why | 5-6 | Bone mineral density (BMD) , PTH ,Ca and P level of the participants were determined and recorded in the Patient’s Information Form for subsequently data analysis. |
| --- | --- | --- | --- | --- |
| Statistical methods | 12 | (*a*) Describe all statistical methods, including those used to control for confounding | 6 | ANOVA variance analysis was adopted for the differences between groups. Pearson correlation analysis was adopted for the correlation between data to calculate the correlation coefficients between data. Logistic binary regression analysis was used for the prediction of independent risk factors for diseases. |
|  |  | (*b*) Describe any methods used to examine subgroups and interactions | N/A |  |
|  |  | (*c*) Explain how missing data were addressed | N/A |  |
|  |  | (*d*) *Cohort study*—If applicable, explain how loss to follow-up was addressed  *Case-control study*—If applicable, explain how matching of cases and controls was addressed  *Cross-sectional study*—If applicable, describe analytical methods taking account of sampling strategy | N/A |  |
|  |  | (*e*) Describe any sensitivity analyses | 6 |  |
| Results | | | | |
| Participants | 13* | (a) Report numbers of individuals at each stage of study—eg numbers potentially eligible, examined for eligibility, confirmed eligible, included in the study, completing follow-up, and analysed | 6-7 | 352 cases were included in the participants after excluding the patients with some underlying diseases, eventually, a total of 245 cases of participants were included in final data analysis. |
|  |  | (b) Give reasons for non-participation at each stage | 7 | 42 cases of the participants once taken anti-osteoporosis medicines or supplementary preparations, such as VD and Ca, in recent period were excluded. Besides, 65 cases of the participants were also excluded due to the deficiency of key data (age, menstrual status, 25(OH)D and BMD). |
|  |  | (c) Consider use of a flow diagram | N/A |  |
| Descriptive data | 14* | (a) Give characteristics of study participants (eg demographic, clinical, social) and information on exposures and potential confounders | 6-7 | The average age of these 245 cases of participants was within 49.5±5.5, of which a total of 167 cases were menopausal women, accounting for 68.2%. The average BMI of the participants was within 23.4±2.8 kg/m2, of which overweight ones (BMI>25.0) accounting 26.9%. |
|  |  | (b) Indicate number of participants with missing data for each variable of interest | N/A |  |
|  |  | (c) *Cohort study*—Summarise follow-up time (eg, average and total amount) | N/A |  |
| Outcome data | 15* | *Cohort study*—Report numbers of outcome events or summary measures over time | 7-10 | Levels of the participants’ 25(OH)D, PTH, Ca and P were showed in tables. |
|  |  | *Case-control study—*Report numbers in each exposure category, or summary measures of exposure |  |  |
|  |  | *Cross-sectional study—*Report numbers of outcome events or summary measures |  |  |
| Main results | 16 | (*a*) Give unadjusted estimates and, if applicable, confounder-adjusted estimates and their precision (eg, 95% confidence interval). Make clear which confounders were adjusted for and why they were included | 7-15 | Details were presented in manuscript. |
|  |  | (*b*) Report category boundaries when continuous variables were categorized |  |  |
|  |  | (*c*) If relevant, consider translating estimates of relative risk into absolute risk for a meaningful time period | N/A |  |

Continued on next page

| Other analyses | 17 | Report other analyses done—eg analyses of subgroups and interactions, and sensitivity analyses |  |  |
| --- | --- | --- | --- | --- |
| Discussion | | | | |
| Key results | 18 | Summarise key results with reference to study objectives | 15-20 | This study considers that VD deficiency in perimenopausal women of Xi'an region is very common and there is no significant correlation between VD deficiency and the level of BMD. This indicates that simple measurement of the levels of bone metabolic markers, such as Ca, P, VD and PTH, can’t really reflect the status of perimenopausal women's BMD in this region. |
| Limitations | 19 | Discuss limitations of the study, taking into account sources of potential bias or imprecision. Discuss both direction and magnitude of any potential bias |  |  |
| Interpretation | 20 | Give a cautious overall interpretation of results considering objectives, limitations, multiplicity of analyses, results from similar studies, and other relevant evidence |  |  |
| Generalisability | 21 | Discuss the generalisability (external validity) of the study results | 21 | This study is of great significance to reasonably guide early clinical intervention of osteoporosis treatment and the occurrence of related fractures. |
| Other information | |  | | |
| Funding | 22 |  | N/A |  |

*Give information separately for cases and controls in case-control studies and, if applicable, for exposed and unexposed groups in cohort and cross-sectional studies.

**Note:** An Explanation and Elaboration article discusses each checklist item and gives methodological background and published examples of transparent reporting. The STROBE checklist is best used in conjunction with this article (freely available on the Web sites of PLoS Medicine at http://www.plosmedicine.org/, Annals of Internal Medicine at http://www.annals.org/, and Epidemiology at http://www.epidem.com/). Information on the STROBE Initiative is available at www.strobe-statement.org.
